# Preliminary Study on Retrograde Recanalization of Radial Artery Occlusion Through Distal Radial Artery Access: a Single-Center Experience

**DOI:** 10.1007/s10557-023-07490-9

**Published:** 2023-07-27

**Authors:** Huanhuan Wang, Cheng Cui, Haiming Liu, Bo Zhang, Tao Tian, Shaodong Ye, Weixian Yang, Jinqing Yuan, Bo Xu, Lijian Gao

**Affiliations:** 1 National Clinical Research Center for Cardiovascular Disease, State Key Laboratory of Cardiovascular Disease, Fuwai Hospital, National Center for Cardiovascular Diseases, Chinese Academy of Medical Sciences and Peking Union Medical College, A 167 Beilishi Road, Xicheng District, Beijing, 100037 China; 2 Yunnan Fuwai Cardiovascular Hospital, Kunming, Yunnan Province China; 3 The People’s Hospital of Dehui City, Dehui, Jilin Province China

**Keywords:** Retrograded recanalization, Radial artery occlusion, Distal transradial access, Experience

## Abstract

**Purpose:**

Radial artery occlusion (RAO) is an unresolved complication after transradial artery (TRA) puncture. The aim of this observational study was to assess the feasibility and safety of retrograde recanalization of RAO through distal transradial access (dTRA).

**Methods:**

From June 2021 to March 2022, 28 consecutive patients with successful puncture and intubation through the dTRA in the anatomical snuffbox and RAO confirmed by angiography were enrolled.

**Results:**

Among the 28 patients, 27 (96.4%) patients with RAO were successfully retrogradely recanalized through the dTRA and successfully underwent coronary angiography or coronary intervention. After the procedure, only 1 (3.7%) patient developed a forearm hematoma, and there were no other bleeding complications or nerve disorders.

**Conclusions:**

DTRA is a safe and feasible approach for retrograded recanalization of RAO, with a high procedure success rate and few complications.

**Supplementary Information:**

The online version contains supplementary material available at 10.1007/s10557-023-07490-9.

## Introduction

Transradial artery access (TRA) has been used for more than 90% [1] of coronary catheterizations (CC) and can reduce the complications related to the approach site and improve the comfort of early walking for patients compared with the transfemoral artery approach [2, 3]. However, TRA also has some complications, such as hematoma, arteriovenous fistula, pseudoaneurysm, osteofascial compartment syndrome, and radial artery occlusion (RAO) [4]. The PROPHET study (Prevention of Radial Artery Occlusion—Patent Hemostasis Evaluation Trial) showed that rates of RAO varied from 5 to 12% and 1.8 to 7% at the 24-h and 30-day follow-ups, respectively [5]. In addition, the failure rates of a second puncture and intubation using the same radial artery (RA) are 3.5% for males and 7.9% for females [6]. Patients with RAO may experience ischemic symptoms, and the TRA approach cannot be used in further catheterizations.

The distal transradial artery (dTRA) is an alternative site for radial artery puncture for CC, including coronary angiography (CAG) and percutaneous coronary intervention (PCI) [7]. Compared with the TRA approach, the dTRA approach has certain advantages, such as faster hemostasis and a lower risk of RAO [8]. The safety and feasibility of dTRA for CC have been demonstrated in a number of studies, but there are few data about RAO recanalization and complete CC through the dTRA approach.

Accordingly, this observational study assessed the success rate of retrograde recanalization of RAO through the dTRA approach.

## Methods

### Study Population

This was a single-center observational study conducted at Fuwai Hospital from June 2021 to March 2022. Patients with a history of TRA were routinely examined for RAO before repeat CC. If the RA pulse was absent, but the distal RA pulse was good, then the dTRA was used first. Finally, 28 consecutive patients who had a successful puncture and intubation of the dTRA at the anatomical snuffbox were enrolled in the study. The patients signed informed consent forms, and the study was reviewed by the Ethics Committee of Fuwai Hospital (2021-1501, Beijing, China).

### Procedural Details

**Fig. 1:**
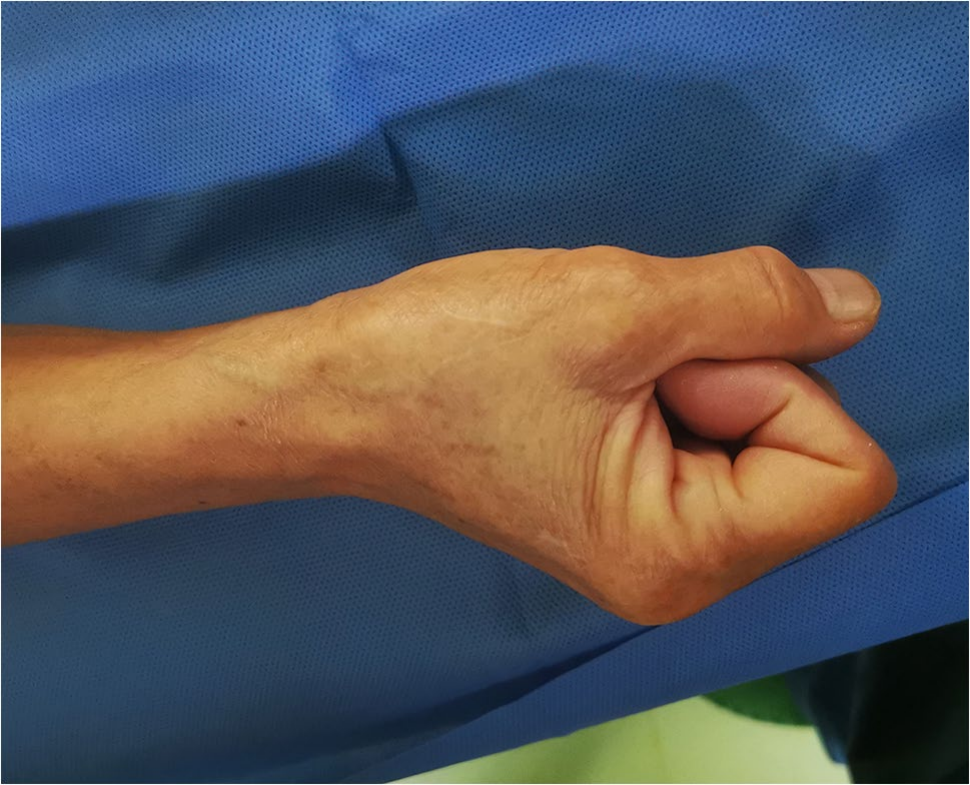
Fully expose the anatomical snuffbox area

**Fig. 2:**
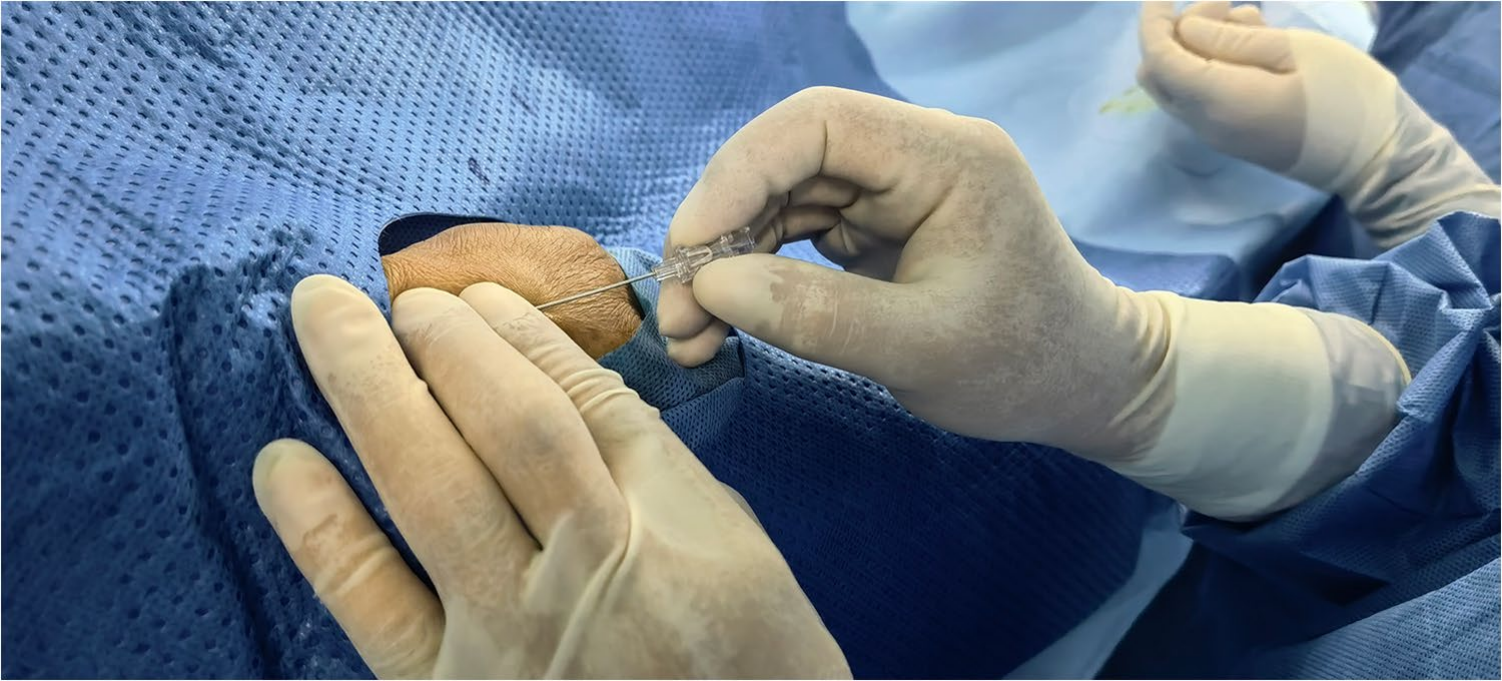
Puncture the distal radial artery with a steel needle at an angle of about 30–45°

**Fig. 3:**
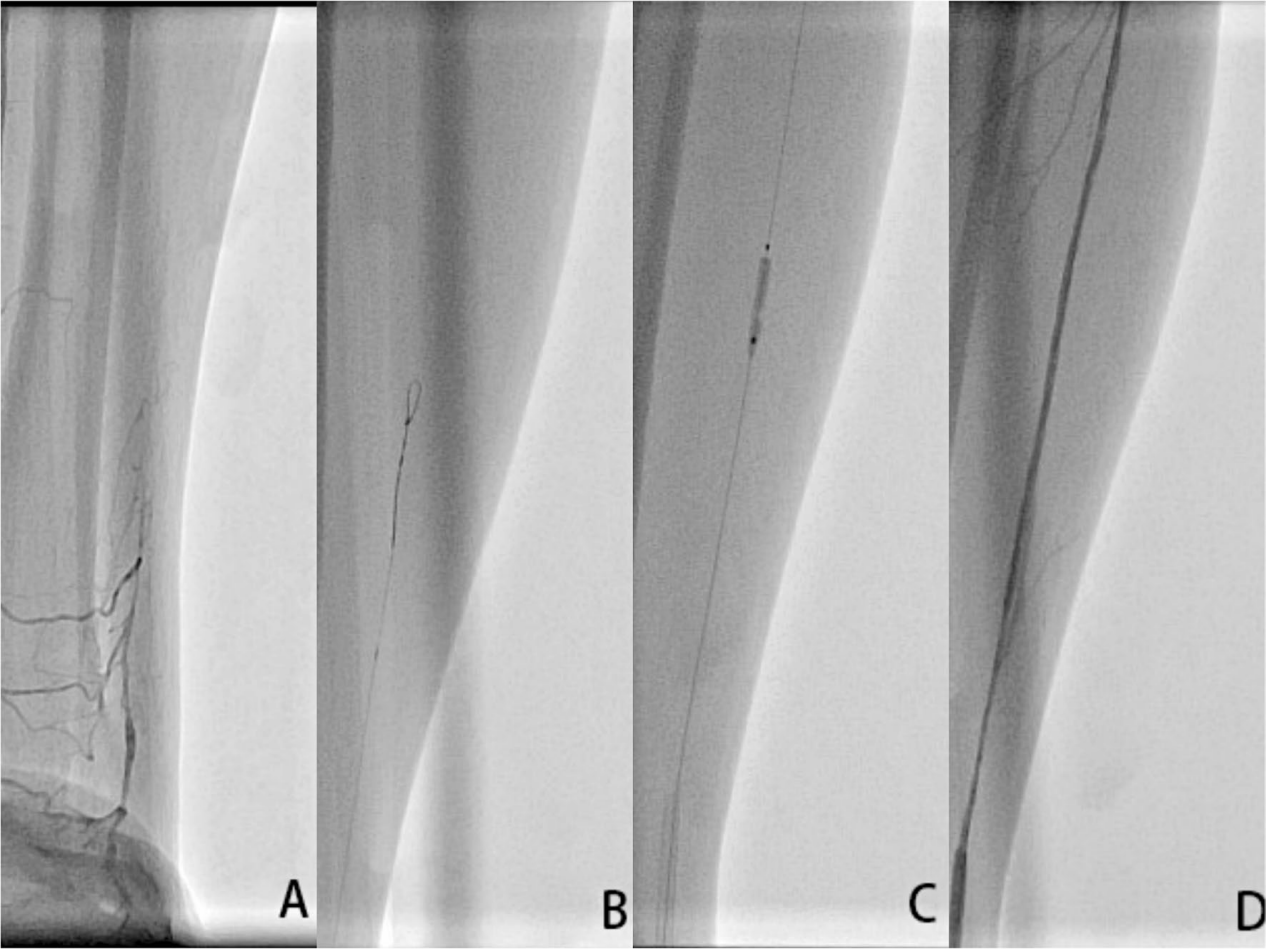
A continuous process of a Pilot 50 guidewire passing through the RAO using the “knuckle” technique and recanalization the RAO

**Fig. 4:**
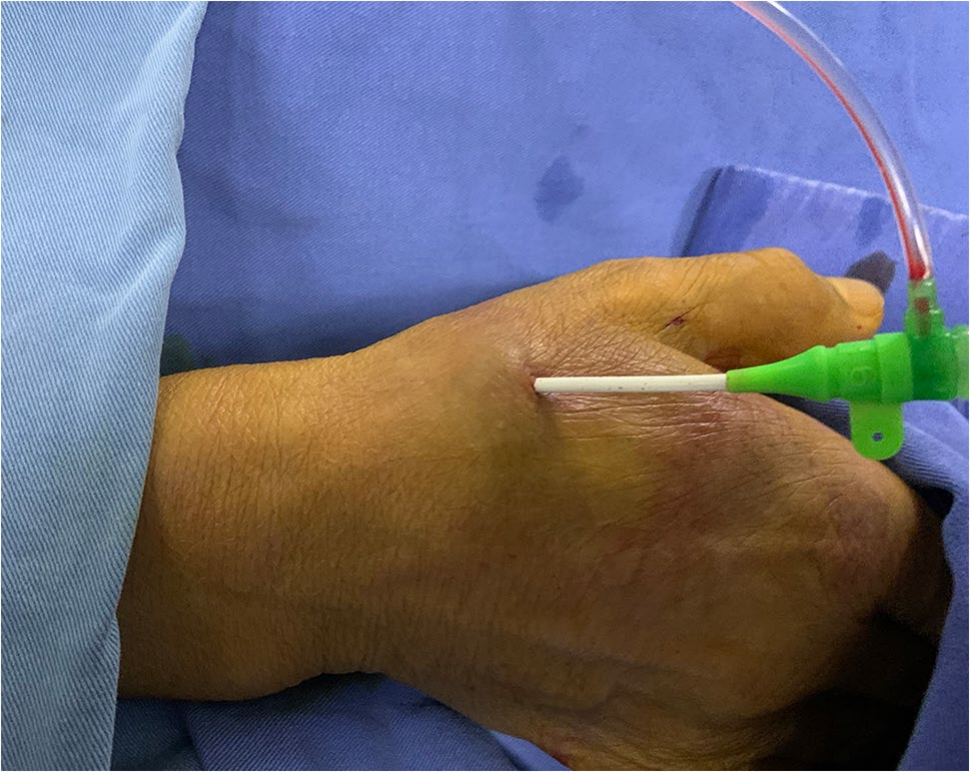
Send the half sheath to the styloid process of the radius

**Fig. 5:**
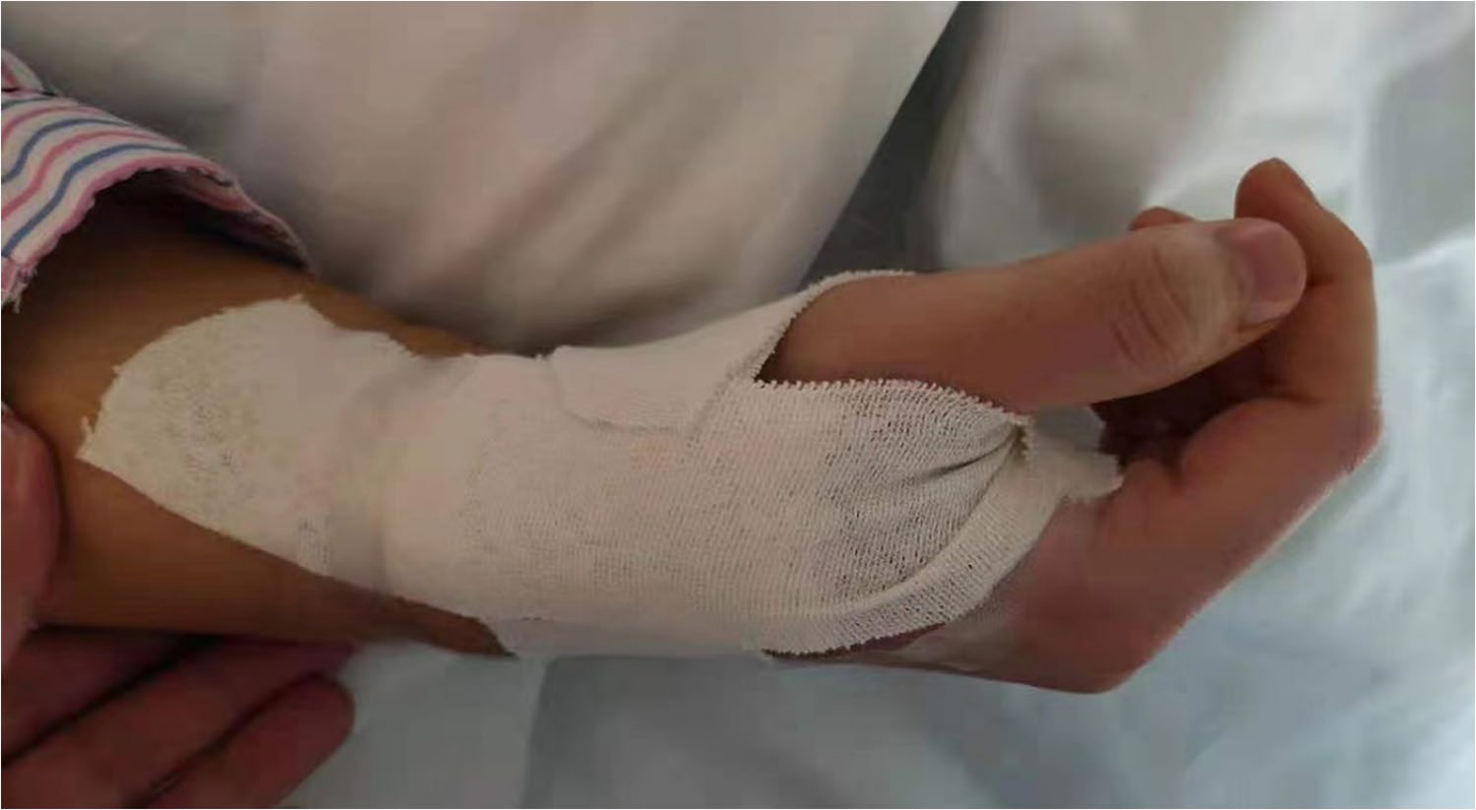
The puncture site in the AS was compressed with gauze and bandage

All CC procedures were performed by Dr. Lijian Gao, who was experienced in the dTRA approach. No Doppler ultrasound was performed prior to puncture. Before puncture, the patient formed a fist to fully expose the anatomical snuffbox (AS) area, which helped in feeling and choosing the site with the strongest pulse for puncture (Fig. 1). Local anesthesia with 2% lidocaine was administered in the AS area. The puncture was performed using a Terumo angiocatheter needle at an angle of approximately 30–45° (Fig. 2). After observing the blood return, angiography was performed using a 5-ml syringe to confirm RAO. Then, a percutaneous coronary intervention (PCI) guidewire was used to attempt to pass through the occluded segment of the RA. When the guidewire was successfully passed through the RA to the brachial artery or subclavian artery, the puncture needle was withdrawn, a balloon with a small diameter should be used demonstrating smooth passage to confirm that it is in the true lumen, and then, the sheath can be inserted. If the occluded segment is at proximal to the RA, the sheath can be inserted only 2–3 cm into the distal radial so that it does not reach the occluded segment, and then, balloon dilation is performed at the site of occluded RA; if the residual stenosis is still severe, progressively larger balloons were used for repeated dilation until the stenosis is relieved (Fig. 3A–D). After the resumption of RA blood flow, we injected 3000 U heparin via the sheath and then inserted a 5-Fr Terumo TIG diagnostic catheter to perform CAG (Fig. 4). For PCI, the sheath was changed to a 6-Fr or 7-Fr sheath, and unfractionated heparin (100 U/kg) was administered to the patients. After the CC procedure, angiography was repeated through the sheath to show the recanalized RA. We then removed the sheath, and the puncture site in the AS was compressed with gauze and a bandage for 4–6 h (Fig. 5).

### Endpoints and Definitions

The primary endpoints of the study were the success rate of retrograde recanalization of RAO through the dTRA approach, defined as blood flow restoration by angiography. Nerve disorders are defined as finger dysfunction and numbness.

### Statistical Analysis

Statistical analysis was performed using SPSS 22.0 statistical software. The measurement data are described as the mean ± standard deviation (*x* ± *s*), and the count data are expressed as a percentage (%).

## Results

### Baseline Patient Characteristics

The baseline characteristics of the study population are shown in Table 1. Among the 28 patients, 20 (71.4%) were male, with an average age of 59 ± 14 years. A total of 27 patients (96.4%) had previous PCI therapy, and 11 patients (39.3%) had a history of PCI ≥ 2 times through the TRA. All of them took dual antiplatelets and statins orally.

**Table 1:** Clinical characteristics

	*N* = 28
Demographic characteristics	
Age; years	59 ± 14
Male gender (%)	20 (71.4)
Mean BMI (kg/m^2^)	26.0 ± 2.6
Co-existing conditions	
Hypertension (%)	19 (67.0)
Diabetes mellitus(%)	10 (35.7)
Dyslipidemia (%)	26 (92.9)
Current smoking (%)	11 (39.3)
Previous PCI (%)	26 (92.9)
Average PCI times	1.8 ± 1.3
0	1 (3.6)
1	16 (57.1)
2	7 (25)
3	1 (3.6)
4	1 (3.6)
5	1 (3.6)
6	1 (3.6)
Antithrombotic drugs	
Aspirin (%)	28 (100)
Clopidogrel(%)	27 (96.4)
Ticagrelor(%)	1 (3.6)
Statin (%)	28 (100)

*BMI* body mass index, *PCI* percutaneous coronary intervention

### Procedural Characteristics

Twenty-seven (96.4%) patients were punctured through the right dTRA, and 1 patient was punctured through the left dTRA. The average number of attempts was 1.6 ± 0.8, and the average puncture time was 4.6 ± 3.4 min. Twenty-two (78.6%) patients required one PTCA guidewire, 5 (17.9%) patients required two PTCA guidewires, and only 1 (3.6%) patient required 3 PTCA guidewires for retrograde RAO. Among them, 27 patients’ guidewires were successfully passed through the occluded RA to the brachial artery or the subclavian artery, and only one failed. One balloon was used in 12 (42.9%) patients, two balloons in 13 (46.4%) patients, and three balloons in 2 (7.1%) patients to sufficiently restore the RAO. The successful rate of retrograde recanalization of RAO was 96.4% (27/28) (Table 2).

**Table 2:** Procedural characteristics of all patients

	*N* = 28
dTRA puncture site	
Left (%)	1 (3.6)
Right (%)	27 (96.4)
Puncture attempt (times, *x* ± *s*)	1.6 ± 0.8
Puncture durations (min, *x* ± *s*)	4.6 ± 3.4
Number of guidewire use (%)	
1	22 (78.6)
2	5 (17.9)
3	1 (3.6)
Successful of guidewire retrograde the RAO (%)	27 (96.4)
Number of balloon use (%)	
1	12 (42.9)
2	13 (46.4)
3	2 (7.1)
Successful of retrograde recanalization of RAO (%)	27 (96.4)

*dTRA* distal transradial access, *RA* radial artery, *RAO* radial artery occlusion

Only one patient failed. This patient was a 44-year-old male who underwent PCI 1 year prior via the right TRA. After successful puncture of the right dTRA, both the Pilot 50 and Fielder XTA guidewires failed to pass through the occluded segment of the RA, and the procedure was then changed to the brachial artery route.

All 27 patients who had a successful retrograded recanalization RAO underwent CAG, including 17 (63.0%) patients who underwent PCI. In fourteen (51.9%) patients, the sheath was changed to a 6-Fr sheath, and in 3 (11.1%), it changed to a 7-Fr sheath. The average duration of angiography and PCI was 13 ± 8 min and 60 ± 30 min, respectively. The average compression hemostasis time was 3.1 ± 0.4 h. Only one patient had a forearm hematoma (3.7%), and there were no other bleeding complications or nerve disorders, such as finger dysfunction or numbness (Table 3).

**Table 3:** Procedural characteristics of patients who had a successful retrograded recanalization of RAO

	*N* = 27
CC type through dTRA	
CAG	10 (37.0)
CAG + PCI	17 (63.0)
PCI sheath size through dTRA	
6 Fr (%)	14 (51.9)
7 Fr (%)	3 (11.1)
Coronary angiography time (min, *x* ± *s*)	13 ± 8
PCI time (min, *x* ± *s*)	60 ± 30
Oppressive time (h, *x* ± *s*)	3.1 ± 0.4
Bleeding adverse events, *n* (%)	
Minor bleeding, *n* (%)	
Mild bleeding, *n* (%)	0
Forearm hematoma, *n* (%)	1 (3.7)
Major bleeding, *n* (%)	0
Nerve disorder, *n* (%)	0

*RA* radial artery, *CC* cardiac catheterization, *dTRA* distal transradial access, *CAG* coronary angiography, *PCI* percutaneous coronary intervention

The baseline characteristics and procedural characteristics of each patient are shown in Table 4.

**Table 4:** Procedural characteristics of each cases

Case	Gender/age (years)	Times of previous TRA CC	Previous TRA CC	No. of guidewires	Guidewires	Guidewire passed through successfully	No. of balloons	Balloon size	Successfully recanalized RAO	CC type	Guide catheter	Target vessel revascularization	Complications
1	F/63	1	4 months	2	Pilot 50, Fielder XTA	Yes	2	Conqueror 1.5 * 15 mm Tazuna 2.0 * 15 mm	Yes	CAG	6 Fr	N/A	No
2	M/58	2	4 months	1	Pilot 50	Yes	1	Conqueror 1.5 * 15 mm	Yes	PCI	6 Fr	PLA	No
3	F/68	1	5 years	1	Pilot 50	Yes	1	Conqueror 2.5 * 15 mm	Yes	PCI	6 Fr	LAD	No
4	M/38	1	5 months	1	Pilot 50	Yes	2	Conqueror 1.5 * 15 mm Sapphire 2.5 * 15 mm	Yes	PCI	6 Fr	LM-LAD	No
5	M/77	6	1 month	1	Pilot 50	Yes	2	Tazuna 1.5 * 15 mm Trek 2.5 * 15 mm	Yes	PCI	6 Fr	LCX, OM1	No
6	M/74	2	12 years	1	Pilot 50	Yes	2	Tazuna 1.5 * 15 mm Pioneer 2.5 * 15 mm	Yes	PCI	6 Fr	LAD, LCX	No
7	F/66	1	1 year	1	Pilot 50	Yes	3	Sprinter 1.5 *15 mm Pioneer 1.5 * 15 mm Conqueror 2.0 * 15 mm	Yes	CAG	6 Fr	N/A	No
8	M/45	2	2 years	1	Pilot 50	Yes	3	Conqueror 1.25 * 15 mm Sapphire 2.5 * 15 mm Sprinter NC 2.5 * 5 mm	Yes	CAG	6 Fr	N/A	No
9	M/34	2	3 years	1	Pilot 50	Yes	2	Sapphire 1.5 * 15 mm Pioneer 2.5 * 20 mm	Yes	PCI	6 Fr	PDA	No
10	M/60	1	9 years	1	Pilot 50	Yes	2	Conqueror 1.5 * 15 mm Tazuna 2.5 * 20 mm	Yes	PCI	6 Fr	LAD	No
11	M/59	1	2 months	2	Pilot 50, Fielder XTA	Yes	2	Tazuna 1.25 * 15 mm Pioneer 2.5*20mm	Yes	PCI	6 Fr	LAD	No
12	M/43	1	3 months	1	Pilot 50	Yes	2	Sapphire 1.5 * 15 mm Tazuna 2.5 * 20 mm	Yes	PCI	7 Fr	LAD	No
13	M/71	3	7 years	1	Pilot 50	Yes	2	Conqueror 1.5 * 15 mm Tazuna 2.5 * 15 mm	Yes	PCI	6 Fr	RCA, LCX	No
14	M/70	1	11 years	1	Pilot 50	Yes	1	Conqueror 2.0 * 20 mm	Yes	CAG	6 Fr	N/A	No
15	F/65	1	2 years	1	Pilot 50	Yes	1	Conqueror 2.0 * 20 mm	Yes	CAG	6 Fr	N/A	No
16	M/61	1	2 years	1	Pilot 50	Yes	1	Conqueror 2.0 * 15 mm	Yes	PCI	6 Fr	LAD	No
17	M.71	1	3 months	2	Anyreach C, Pilot 50	Yes	2	Conqueror 2.0 * 15 mm Conqueror 2.0 * 20 mm	Yes	PCI	6 Fr	LCX	No
18	F/79	1	2 years	1	Pilot 50	Yes	1	Pioneer 2.0 * 15 mm	Yes	CAG	6 Fr	N/A	No
19	M/39	1	1 year	1	Pilot 50	Yes	1	Pioneer 2.5 * 15 mm	Yes	CAG	6 Fr	RCA	No
20	M/44	1	1 year	2	Pilot 50, Fielder XTA	No	NA	NA	No	CAG	6 Fr	N/A	No
21	F/66	1	1 year	1	Pilot 50	Yes	2	Sapphire 1.5 * 15 mm Conqueror 2.5 * 15 mm	Yes	PCI	6 Fr	LCX	No
22	M/48	2	8 years	3	Pilot 50 * 2, Fielder XTA	Yes	2	Tazuna 1.5 * 15 mm Tazuna 2.5 * 15 mm	Yes	CAG	6 Fr	N/A	No
23	M/76	2	7 years	2	Pilot 50, Pilot 150	Yes	2	Tazuna 1.5 * 15 mm Tazuna 2.5 * 15 mm	Yes	CAG	6 Fr	LAD	Forearm hematoma
24	F/35	1	12 days	1	Pilot 50	Yes	1	Pioneer 2.5 * 15 mm	Yes	PCI	7 Fr	LAD	No
25	F/69	0	4 years	1	Pilot 50	Yes	1	Pioneer 2.0 * 15 mm	Yes	CAG	6 Fr	N/A	No
26	M/57	5	N/A	1	Pilot 50	Yes	1	Pioneer 2.0 * 15 mm	Yes	PCI	6 Fr	LAD	No
27	M/69	4	6 days	1	Pilot 50	Yes	1	Conqueror 1.5 * 15 mm	Yes	CAG	7 Fr	LM-LAD	No
28	M/40	2	9 months	1	Pilot 50	Yes	1	Hoper 2.5 * 20 mm	Yes	PCI	6 Fr	LAD, LCX	No

*TRA* transradial access, *CC* cardiac catheterization, *RAO* radial artery occlusion

## Discussion

The main finding of this observational clinical study was the high acceptable success rate of the dTRA approach for retrograde recanalization of RAO caused by previous cardiac catheterization. Thus, the dTRA approach is considered safe and feasible for this purpose.

Currently, TRA is recommended by ESC guidelines as the standard approach for CAG and PCI [9]. However, RAO is still an unwell-resolved complication of the TRA approach, and with the rapid increase in the use of TRA, the number of RAO patients is also rising [10, 11]. Factors associated with RAO include body mass index, diabetes mellitus, female sex, repeated TRA, large sheath size, anticoagulant usage, long operation, and compression time [11, 12]. Most RAO patients will not experience hand ischemia owing to the dual vascular supply of the palmar arch. Thus, the incidence of RAO may be underestimated in the real world [13]. However, for some patients, RAO still affected the physical activity of the arm or caused numbness. More importantly, RAO limits future utilization of the RA, including repeated CAG and PCI, for establishing dialysis access and for use in bypass graft in coronary artery bypass graft (CABG) surgery [8]. Therefore, finding a way to recanalize RAO has become very important.

The dTRA was first reported by Kiemeneij as an alternative access for CC in 2017 [7]. Compared with the TRA approach, the dTRA approach has advantages in terms of patient comfort, faster hemostasis, and a lower risk of RAO, which has attracted the attention of cardiologic interventionalists [14, 15]. It was found that the RAO can be opened through the dTRA. Our team reported successful angioplasty via the dTRA in recanalizing the right RAO of a 68-year-old man with a history of PCI, and a 3-month follow-up vascular ultrasound showed that the RA had smooth blood flow and no stenosis [16]. Some small-scale case series have suggested that dTRA is safe and feasible for retrograde recanalization of RAO, with a success rate of approximately 88–93% [17, 18]. In the present study, the success rate was 96.7% with very low complications, and successful patients underwent CAG and PCI. The high success rate is important to maintain radial access for future procedures.

There are some technical considerations recommended for recanalizing an RAO. First, successful dTRA puncture in the AS area is crucial. The pulsation of the distal RA in the AS area must be palpable or ultrasound-guided to improve the puncture success rate. Second, after successful puncture, a guidewire used for coronary balloon angioplasty is used to cross the RAO. The procedure was similar to PCI. The “knuckle” technique can be used to retrograde the guidewire to the brachial artery (Fig. 3B). In this study, successful retrograded RAO was achieved in 23 patients with Pilot 50, which is a hydrophilic PTCA guidewire. For the other patients, in whom Pilot 50 failed to be applied, 3 patients were changed to Fielder XTA, which is a hydrophilic guidewire, and the other patient was changed to Pilot 150, which has greater hardness. Only one patient failed with both Pilot 50 and Fielder XTA. In most patients, the occluded RA can be passed through by using a single hydrophilic guidewire. If it fails, it can be changed to another hydrophilic guidewire. Because RAO is different from coronary artery occlusion, the occlusion site is mainly thrombosis, and there is no need for a high tip-load guidewire such as Confianza Pro 12 or Gaia Third. Our experience was that the combined application of hydrophilic guidewire will improve the success rate of retrograde guidewire passage. Third, a small-diameter balloon size (1.25–1.5 mm) should be used to check if it is in the true vascular lumen; then, a balloon angioplasty is performed repeatedly at the occluded segment of RA with a larger balloon (diameter of 2.0–2.5 mm). Our principle of management is to use balloon dilation from small to large to achieve satisfactory dilation of the occluded radial artery. After it is confirmed that the sheath can be placed in the vascular lumen, subsequent CAG and PCI can be performed smoothly.

In this study, only one patient had a hematoma after the procedure. Although the experience with this technique is rather limited, there are several suggestions to improve outcomes. First, the surgeon should be experienced in dTRA, especially with a high rate of successful access. Ultrasound guidance can improve the successful puncture rate [19]. Second, when the guidewire successfully passes the RAO, a balloon with a small diameter should be used demonstrating smooth passage to confirm that it is in the true lumen, and then, the sheath can be inserted, which can avoid vascular injury and reduce the risk of bleeding. If the occluded segment is proximal to the RA, the sheath can be inserted only 2–3 cm into the distal radial so that it does not reach the occluded segment. Whether the sheath or balloon dilation is advanced depends on the location of the occluded segment of the RA. Finally, after CAG and PCI, radial arteriography should be performed to check whether there is a serious RA injury.

The long-term result of retrograde recanalization of RAO remains unclear. Small studies showed that the patency rates at 3, 6, and 12 months were 48.7%, 43.6%, and 35.9%, respectively. Balaban and Elevli showed that for 14 patients given drug-coated balloon (DCB) treatment after angioplasty, the patency rate was only 33.4% at the 1-month follow-up [16]. Such a low patency rate may be due to the different pathological mechanisms between RAO and coronary atherosclerosis [20]. No stent was used in the radial artery because the long-term patency rate was uncertain, and it interfered with future catheterizations.

## Limitations

First, this is a single-center observational study with a small sample size, and not all the RAO patients were screened and had their data recorded. Therefore, we do not have data on how many patients with RAO were screened and found unacceptable. The data we are currently collecting can answer this question in the future. Second, not all patients underwent vascular ultrasound examination before and after the procedure, and the long-term patency rate of the RA is unknown. Third, intraluminal imaging was not performed in RA, which may be helpful for understanding the structure of RA for follow-up treatment. In the future, how to ensure the long-term patency rate of RA may be our research direction.

## Conclusions

DTRA is a safe and feasible approach for retrograded recanalization of RAO, with a high procedure success rate and few complications.

## Supplementary Information

ESM 1 (DCM 124441 kb)

ESM 2 (DCM 2581 kb)

ESM 3 (DCM 34582 kb)

ESM 4 (DCM 25878 kb)

ESM 5 (PDF 63 kb)

ESM 6 (PDF 69 kb)

## Data Availability

The data that support the findings of this study are available on request from the corresponding author. The data are not publicly available due to privacy or ethical restrictions.

## Abbreviations

CC: Cardiac catheterization
TRA: Transradial access
RAO: Radial artery occlusion
RA: Radial artery
dTRA: Distal transradial access
CAG: Coronary angiography
PCI: Percutaneous coronary intervention
CAD: Coronary artery disease
CABG: Coronary artery bypass grafting
PTCA: Percutaneous transluminal coronary angioplasty

## Code Availability

Statistical analysis was performed using SPSS 22.0 statistical software.

## References

[1] Wang H, Gao Z, Yuan J, et al. Association of body mass index with mortality in Chinese patients after percutaneous coronary intervention: a large single-center data. Cardiovasc Ther. 2017;35(4):1–8. 10.1111/1755-5922.12271 28467641

[2] Rao SV, Dharma S. 25 years of transradial intervention: looking back and anticipating what is ahead. JACC Cardiovasc Interv. 2017;10(22):2266–8. 29169495 10.1016/j.jcin.2017.08.047

[3] Agostoni P, Biondi-Zoccai GG, de Benedictis ML, Rigattieri S, Turri M, Anselmi M, et al. Radial versus femoral approach for percutaneous coronary diagnostic and interventional procedures; systematic overview and meta-analysis of randomized trials. J Am Coll Cardiol. 2004;44(2):349–56. 15261930 10.1016/j.jacc.2004.04.034

[4] Coomes EA, Haghbayan H, Cheema AN. Distal transradial access for cardiac catheterization: a systematic scoping review. Catheter Cardiovasc Interv. 2020;96(7):1381–9. 31785083 10.1002/ccd.28623

[5] Pancholy S, Coppola J, Patel T, Roke-Thomas M. Prevention of radial artery occlusion-patent hemostasis evaluation trial (PROPHET study): a randomized comparison of traditional versus patency documented hemostasis after transradial catheterization. Catheter Cardiovasc Interv. 2008;72(3):335–40. 18726956 10.1002/ccd.21639

[6] Sakai H, Ikeda S, Harada T, Yonashiro S, Ozumi K, Ohe H, et al. Limitations of successive transradial approach in the same arm: the Japanese experience. Catheter Cardiovasc Interv. 2001;54(2):204–8. 11590685 10.1002/ccd.1268

[7] Kiemeneij F. Left distal transradial access in the anatomical snuffbox for coronary angiography (ldTRA) and intervention (ldTRI). Eurointervention. 2017;13(7):851–7. 28506941 10.4244/EIJ-D-17-00079

[8] Corcos T. Distal radial access for coronary angiography and percutaneous coronary intervention: a state-of-the art review. Catheter Cardiovasc Interv. 2019;93(4):639–44. 30536709 10.1002/ccd.28016

[9] Neumann FJ, Sousa-Uva M, Ahlsson A, Alfonso F, Banning AP, Benedetto U, et al. ESC/EACTS Guidelines on myocardial revascularization. Eur Heart J. 2019;40(2):87–165. 30165437 10.1093/eurheartj/ehy394

[10] Hahalis G, Aznaouridis K, Tsigkas G, Davlouros P, Xanthopoulou I, Koutsogiannis N, et al. Radial artery and ulnar artery occlusions following coronary procedures and the impact of anticoagulation: ARTEMIS (Radial and Ulnar Artery Occlusion Meta-AnalysIS) systematic review and meta-analysis. J Am Heart Assoc. 2017;6(8):e005430. 28838915 10.1161/JAHA.116.005430 PMC5586412

[11] Avdikos G, Karatasakis A, Tsoumeleas A, Lazaris E, Ziakas A, Koutouzis M. Radial artery occlusion after transradial coronary catheterization. Cardiovasc Diagn Ther. 2017;7(3):305–16. 28567356 10.21037/cdt.2017.03.14 PMC5440258

[12] Garg N, Madan BK, Khanna R, Sinha A, Kapoor A, Tewari S, et al. Incidence and predictors of radial artery occlusion after transradial coronary angioplasty: doppler-guided follow-up study. J Invasive Cardiol. 2015;27(2):106–12. 25661763

[13] Ali S, Abdullah MS, Abdelrahman K, Ali A, Faisal F, Ali A. Total radial artery occlusion following transradial access: complete recanalization via the anatomical snuffbox. Methodist Debakey Cardiovasc J. 2020;16(4):314–7. 33500761 10.14797/mdcj-16-4-314 PMC7812849

[14] Sgueglia GA, Giorgio AD, Gaspardone A, Babunashvili A. Anatomic basis and physiological rationale of distal radial artery access for percutaneous coronary and endovascular procedures. JACC Cardiavasc Interv. 2018;11(20):2113–9. 10.1016/j.jcin.2018.04.045 30336816

[15] Al-Azizi KM, Grewal V, Gobeil K, Maqsood K, Haider A, Mohani A, et al. The left distal transradial artery access for coronary angiography and intervention: a US experience. Cardiovasc Revasc Med. 2019;20(9):786–9. 30413346 10.1016/j.carrev.2018.10.023

[16] Song K, Wang H, Li H, Cui Y, Gao L. A case of using the distal radial artery to open an occluded radial artery. JACC Case Rep. 2020;2(15):2432–3. 34317188 10.1016/j.jaccas.2020.10.026 PMC8305079

[17] Balaban Y, Elevli MG. It is both possible and safe to perform coronary angiography through the same radial artery, after retrograde recanalization of radial artery occlusion, following a previous coronary angiography. J Interv Cardiol. 2018;31(6):957–63. 29855079 10.1111/joic.12524

[18] Yuan F, Zuo K, Yang Y, Gao Y, Liu L, Ding X, et al. Distal transradial access: a safe and feasible approach for coronary catheterization in cases of total radial artery occlusion. Journal of Cardiovascular Translational Research. J Cardiovasc Transl Res. 2022;15(5):1203–11. 35334079 10.1007/s12265-022-10238-9

[19] Hadjivassiliou A, Kiemeneij F, Nathan S, et al. Ultrasound-guided access to the distal radial artery at the anatomical snuffbox for catheter-based vascular interventions: a technical guide. EuroIntervention. 2021;16(16):1342–8. 31380781 10.4244/EIJ-D-19-00555 PMC9724968

[20] Kitrou P, Parthipun A, Diamantopoulos A, Padayachee S, Karunanithy N, Ahmed I, et al. Paclitaxel-coated balloons for failing peripheral bypass grafts: the BYPACS study. J Cardiovasc Surg (Torino). 2014;55(2):217–24. 24670829

