# Supplementary material for: Preliminary Study on Retrograde Recanalization of Radial Artery Occlusion Through Distal Radial Artery Access: a Single-Center Experience
Source: Cardiovasc Drugs Ther. 2023 Jul 27;38(6):1303–13. doi: 10.1007/s10557-023-07490-9 (PMC11680607; doi:10.1007/s10557-023-07490-9)
Supplement: Supplementary file 5 — (PDF 63 kb) [file 10557_2023_7490_MOESM5_ESM.pdf]

INVOICE

Invoice# SK61K3KKN

Balance Due
CNY0.00

美刊在线（北京）信息咨询有限公司
China

Invoice Date :	2023-03-28
Terms :	Due On Receipt
Due Date :	2023-03-28
Submission :	K61K3KKN
Word Count :	2563
Title :	Analysis the efficacy and safety of coronary catheterization through distal transradial access: A s…
Discounts Applied :	$40 (52E394)

Bill To
Huanhuan Wang
Dr. Huanhuan Wang

#	Item & Description	Rate	Discount	Amount
1	Standard Editing<br>Standard Editing	1,358.99	262.00	1,096.99

Sub Total	1,096.99
**Total**	**CNY1,096.99**
Payment Made	(-) 1,096.99
**Balance Due**	**CNY0.00**

AJE INVOICE
