# Supplementary material for: Preliminary Study on Retrograde Recanalization of Radial Artery Occlusion Through Distal Radial Artery Access: a Single-Center Experience
Source: Cardiovasc Drugs Ther. 2023 Jul 27;38(6):1303–13. doi: 10.1007/s10557-023-07490-9 (PMC11680607; doi:10.1007/s10557-023-07490-9)
Supplement: Supplementary file 6 — (PDF 69 kb) [file 10557_2023_7490_MOESM6_ESM.pdf]

INVOICE

Invoice# SZF99CH7Y

Balance Due
CNY0.00

601 W Main St, Ste 102
Durham, North Carolina, 27701, USA
Tax ID: 412141424

Invoice Date :	30 Aug 2022
Submission :	ZF99CH7Y
Word Count :	2470
Title :	Preliminary study on recanalize the occluded radial artery through distal radial artery access: A s⋯

Bill To
Wang Huanhuan
A 167th Beilishi Road, Xicheng District
A 528th Shahe North Road, Wuhua District
Beijing
100037 Beijing
China
Dr. Huanhuan Wang
+861068351786

#	Item & Description	Base Price	Amount
1	Standard Editing<br>Standard Editing	1,604.75	1,604.75

Sub Total	1,604.75
**Total**	**CNY1,604.75**
Payment Made	(-) 1,604.75
**Balance Due**	**CNY0.00**

AJE
INVOICE

请注意：本电子收据创建时间显示的是美国东部时间，该时间比您的当地时间可能晚12个（春夏）或13个小时（秋冬）。
